# High-speed optical coherence tomography angiography for the measurement of stimulus-induced retrograde vasodilation of cerebral pial arteries in awake mice

**DOI:** 10.1117/1.NPh.7.3.030502

**Published:** 2020-09-10

**Authors:** Paul Shin, Jin-Hui Yoon, Yong Jeong, Wang-Yuhl Oh

**Affiliations:** a Korea Advanced Institute of Science and Technology, Department of Mechanical Engineering, Daejeon, Republic of Korea; b Korea Advanced Institute of Science and Technology, KI for Health Science and Technology, Daejeon, Republic of Korea; c Korea Advanced Institute of Science and Technology, Department of Bio and Brain Engineering, Daejeon, Republic of Korea

**Keywords:** retrograde vasodilation, functional hyperemia, optical coherence tomography, angiography, awake imaging

## Abstract

**Significance:** Having a clear understanding of functional hyperemia is crucial for functional brain imaging and neurological disease research. Vasodilation induced by sensory stimulus propagates from the arterioles to the upstream pial arteries in a retrograde fashion. As retrograde vasodilation occurs briefly in the early stage of functional hyperemia, an imaging technique with a high temporal resolution is required for its measurement.

**Aim:** We aimed to present an imaging method to measure stimulus-induced retrograde vasodilation in awake animals.

**Approach:** An imaging method based on optical coherence tomography angiography, which enables a high-speed and label-free vessel diameter measurement, was developed and applied for the investigation.

**Results:** The propagation speed of retrograde vasodilation of pial artery was measured in awake mice. Other characteristics of functional hyperemia such as temporal profile and amplitude of the vascular response were also investigated.

**Conclusions:** Our results provide detailed information of stimulus-induced hemodynamic response in the brain of awake mice and suggest the potential utility of our imaging method for the study of functional hyperemia in normal and diseased brain.

## 1 Introduction

Neurovascular coupling, a close interplay between neural activity and cerebral blood flow (CBF), regulates the blood supply of corresponding brain regions. The regulation is mediated by dilation and/or constriction of cerebral vessels. In order to effectively perfuse blood upon stimuli, the dilation of arterioles should be accompanied by the dilation of upstream pial arteries.^1^^–^^3^ Dilation of arterioles induced by neural activation propagates to the pial arteries in a retrograde fashion.^2^ A detailed mechanism of this coordinated vascular adjustment remains elusive; however, accumulating evidence indicates that such may be due to the signal transduction through endothelial and smooth muscle cell gap junctions in vessel walls.^4^^,^^5^ This hemodynamic response function of the brain is disrupted in pathological conditions. For example, attenuation of the CBF increase induced by a sensory stimulus has been reported in Alzheimer’s disease, hypertension, and ischemic stroke.^1^ The entire hemodynamic response process, including the amplitude of the response, could be affected by various pathological factors.

Having a clear understanding of functional hyperemia both in normal and pathological conditions is critical for the interpretation of the signals of hemodynamically based imaging systems, such as blood-oxygen-level-dependent signal in functional magnetic resonance imaging.^6^ However, rapid hemodynamic changes, such as retrograde vasodilation, have not been properly investigated because of the insufficient temporal resolution of conventional imaging techniques.^7^^,^^8^ Although a high-speed optical intrinsic signal imaging (OISI) technique, with a frame rate of several tens of Hertz, could provide an indirect measurement of the propagation of vasodilation by estimating the changes in arterial hemoglobin concentration,^9^ a direct imaging technique with a high spatiotemporal resolution is required for the precise measurement of retrograde vasodilation.

Two-photon laser scanning microscopy (TPLSM) was utilized for direct measurement of vessel diameter by scanning the focal spot of a laser across the width of the vessel.^10^^,^^11^ However, the point scanning geometry of TPLSM allows assessment of only the vessel segments within a narrow focal depth.^12^^–^^14^ Recently, optical coherence tomography angiography (OCTA) has been introduced in various functional imaging studies.^15^^–^^17^ OCTA provides a high spatial and temporal resolution three-dimensional (3-D) imaging of cerebral microvasculature and facilitates detailed hemodynamic analysis, taking into account the 3-D vessel geometry.^17^ In addition, OCTA enables accurate measurement of the vessel diameter without using any contrast agent injection, which makes this technology well suited for awake imaging studies and longitudinal imaging studies.^18^^,^^19^ Thus, in this study, we aimed to present the potential of OCTA for the measurement of stimulus-induced retrograde vasodilation in the awake mouse brain.

## 2 Materials and Methods

### 2.1 Animal Preparation

**Fig. 1:**
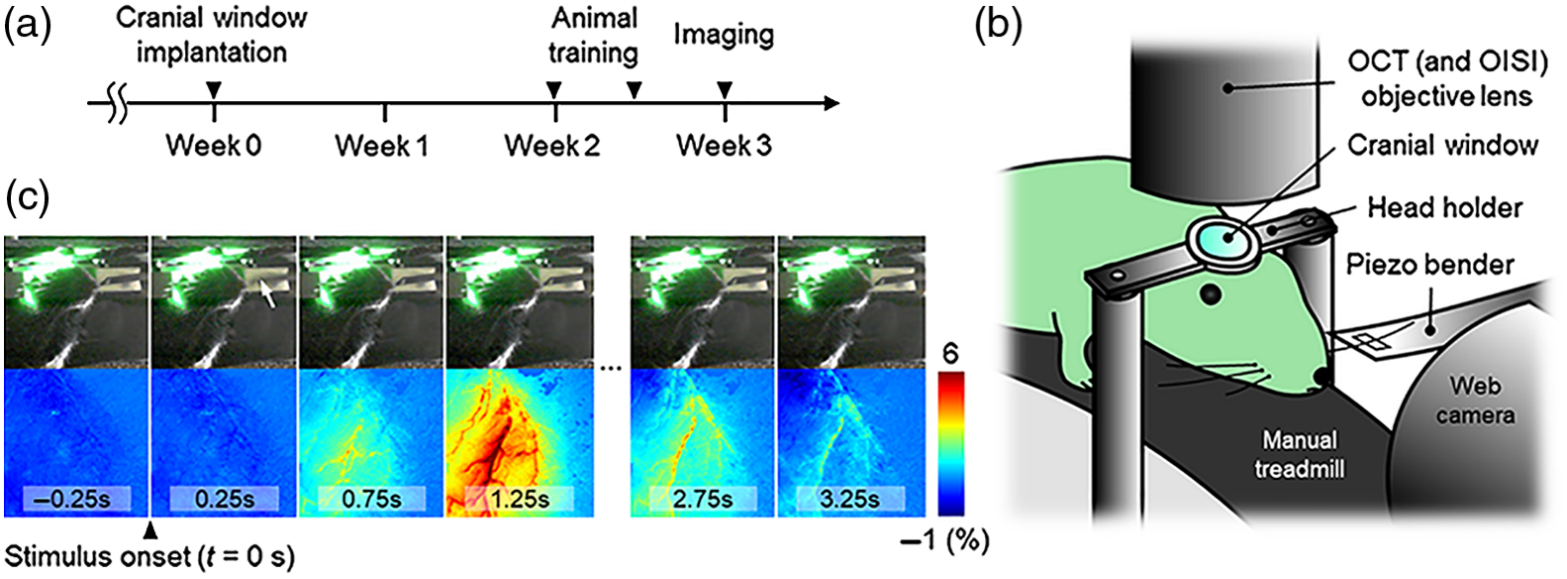
(a) Experimental timeline. (b) The imaging setup for awake imaging. (c) Web camera images of an awake mouse acquired during OISI (top row), and OISI images of the mouse cranial window simultaneously acquired with the web camera images showing hemodynamic response induced by the 0.3-s whisker stimulus (bottom row) (Video 1, MP4, 2.3 MB [URL: https://doi.org/10.1117/1.NPh.7.3.030502.1]).

All animal experimental procedures were approved by the Institutional Animal Care and Use Committee of Korea Advanced Institute of Science and Technology (KA2020-07). Male C57BL/6N mice (n=4) were used for this study. The experimental timeline is shown in Fig. 1(a). A chronic cranial window was implanted on the right barrel cortex, as previously described.^20^ A custom-made head holder was positioned over the window to minimize motion artifacts during imaging. After 2 weeks of recovery, all animals were trained for awake imaging. Specifically, after brief anesthesia with isoflurane (3%, v/v), the mouse was fixed by the head holder to two stationary vertical shafts and left to rest or move freely forward and backward on a treadmill. All whiskers at the left side of the animal were trimmed except E1 whisker, which was fixed at the tip of a piezo bender. The bender vibrated the whisker with a frequency of 10 Hz and with an amplitude of 2 to 3 mm at 10 mm from the mystacial pad. Of the four mice, one was used for the additional imaging session with an intraperitoneal administration of a mixture of ketamine and xylazine (100 and 10  mg/kg, respectively) to investigate the effects of anesthesia on hemodynamic response. During the experiment under anesthesia, body temperature was maintained at 36.5°C to 37.5°C. Heart rate and arterial oxygen saturation were also continuously monitored.

### 2.2 Imaging Setup and Procedures

Figure 1(b) shows the imaging setup for awake imaging. A swept-source OCT system with an A-line rate of 240 kHz was used, as previously described.^17^ The center wavelength of a wavelength-swept laser was 1.3  μm, and the axial and transverse resolutions (full-width at half-maximum) of the system are both 10  μm. An OISI system was designed to share the objective lens with OCT. For OISI, a green light emitting diode mounted with a band-pass filter (530±5  nm) was used as a light source for the measurement of the changes in total hemoglobin concentration.^9^ A web camera was positioned facing the animals to observe their behavior.

For OISI, two-dimensional (2-D) images were continuously acquired using a charge coupled device (CCD) camera for 10.5 s. During the acquisition, a 0.3-s whisker stimulus was applied 1.66 s after the onset of the acquisition where the stimulus consisted of a series of three sinusoidal whisker vibrations. A series of OISI images were generated by computing the fractional intensity difference between the baseline and response images. Seven sequential OISI images were averaged to produce a single averaged OISI image, and the resulting temporal resolution of the OISI was 0.25 s. Stimulus-induced changes in total hemoglobin concentration were observed in the OISI images of an awake resting mouse [Fig. 1(c) and Video 1].

**Fig. 2:**
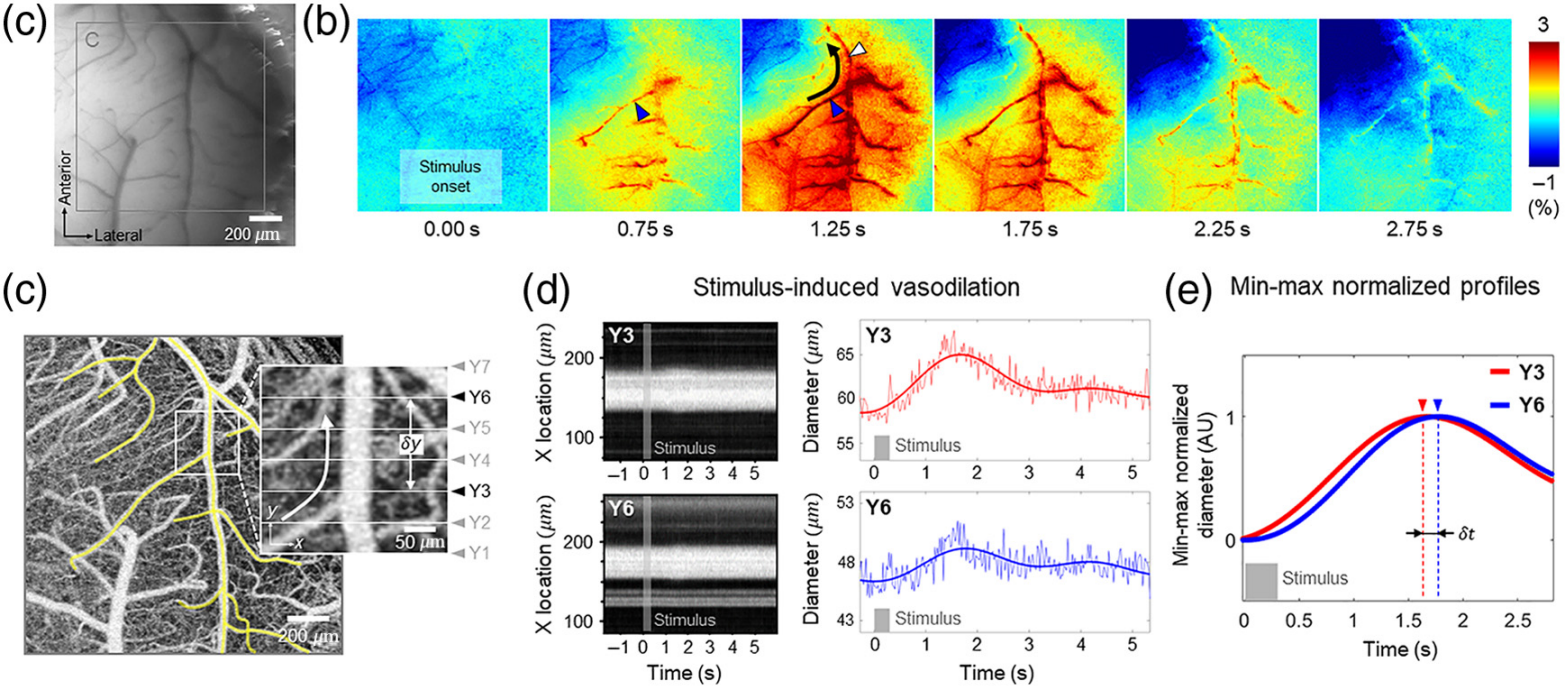
(a) A CCD image of the mouse cranial window. (b) OISI images of the region shown in (a). The black arrow indicates the direction of the propagation. (c) A depth-projected OCT angiogram of the region indicated by the gray square in (a) (0 to 400  μm from the cortical surface, maximum projection). A magnified view of the region where OCTA-2 was performed is shown (white square). The white arrow indicates the direction of the propagation of total hemoglobin response, which was identified by OISI. (d) Left column: depth-projected OCT angiograms continuously acquired at Y3 and Y6 in (c). The stimulus application is indicated by the gray-shaded area. Right column: the average time courses of stimulus-induced change in vessel diameter measured at Y3 and Y6. The thick and thin lines indicate a time course with and without low-pass filtering, respectively. (e) Two average time courses in (d) are overlapped; low-pass filtering and minimum–maximum normalization were performed for each time course (Video 2, MP4, 2.9 MB [URL: https://doi.org/10.1117/1.NPh.7.3.030502.2]).

We performed two different OCTA protocols as follows: OCTA-1, which was for identifying the geometric structure of pial vessels, and OCTA-2, which was for measuring rapid hemodynamic changes. For OCTA-1, a 3D-OCT angiogram was obtained at the region where the OISI was performed with a field of view (FOV) of 1.5  mm×1.5  mm. The 3D-OCT angiogram was composed of 1000 A-lines and 800 B-scans in the x and y directions, respectively. Each 2-D cross-sectional OCT angiogram was obtained by calculating the speckle decorrelation between a pair of consecutive OCT intensity cross-section images acquired at the same y location.^17^^,^^21^ For OCTA-2, a series of 3-D intensity OCT data, where each volume consisting of 7 B-scans (400 A-lines/B-scan) imaged with a 50-μm spacing over an FOV of 300  μm×300  μm, were continuously acquired for 7.58 s, with a volumetric rate of 86 volumes per s. Seven different locations across the width of a pial artery that showed a prominent response in the OISI images were scanned [indicated by white horizontal lines in the magnified inset in Fig. 2(c)]. A 3D-OCT angiogram was obtained by calculating the speckle decorrelation between two consecutive 3D-OCT intensity images, and the resulting temporal resolution of OCTA was 0.023 s. A whisker stimulus was applied for 0.3 s, which was initiated 1.66 s after the start of the image acquisition. To measure the average hemodynamic response, OCTA-2 was performed repeatedly (30 times). The entire imaging procedure was completed in 40 min.

### 2.3 Data Analysis

By monitoring with the web camera, all imaging was performed when the animal was at rest without any movement to minimize motion induced hemodynamic variations. Video 2 shows representative cross-sectional OCT images of the awake mouse brain.

For each angiogram acquired by OCTA-2, the average intensity projection was performed in the depth-direction (0 to 300  μm from the cortical surface). Consequently, a series of 2-D *en face* OCT angiograms were obtained. Vessel diameter was measured as the width of the vessel from the depth-projected OCT angiograms, as previously described.^17^

To reduce the effect of arterial pulsatility, low-pass filtering with a cutoff frequency of 3 Hz was performed for each time course before the averaging procedure. The selected cutoff frequency was based on the normal heart rate of a mouse, which ranges from 400 to 700 beats per minute.

## 3 Results

A representative CCD image of the mouse cranial window and the corresponding OISI images are shown in Figs. 2(a) and 2(b). As shown in the OISI images, the increasing total hemoglobin response propagated retrogradely from the arteriole (blue arrowhead) to the upstream pial artery (white arrowhead). The propagation speed of total hemoglobin response was obtained by measuring the time delay between the OISI signals at different locations with a temporal resolution of 0.25 s (Fig. S1 in the Supplementary Material). The mean propagation speed of total hemoglobin response for the four mice was ∼1.6  mm/s.

A depth-projected image of the 3D-OCT angiogram of the region indicated by the gray square in Fig. 2(a) is shown in Fig. 2(c), which was obtained by OCTA-1. A pial artery and its first-order branches are shaded in yellow. Temporal series of 3D-OCT angiograms (OCTA-2) were continuously acquired at the region where the retrograde propagation of total hemoglobin response was observed [magnified inset in Fig. 2(c)]. A detailed procedure of selecting the region of interest is described in Fig. S2 in the Supplementary Material. Each 3D-OCT angiogram consisted of seven cross-sectional OCT angiograms that were acquired at equally spaced y locations (indicated as white lines in the magnified inset). To measure the propagation of vasodilation, pial artery diameters were measured at two different y locations (Y3 and Y6), with a distance of δy. Although the distance δy was set to 150  μm in Fig. 2(c), as an example, δy could be adjusted to multiples of the minimum distance, i.e., 50  μm. A single segment of pial artery with no branch between the measurement locations (Y3 and Y6) was used for the propagation measurement. The left column in Fig. 2(d) with the two depth-projected OCT angiograms acquired for 7.58 s at Y3 and Y6, respectively, shows that the stimulus clearly increased the vessel diameters. The right column in Fig. 2(d) shows the average time course of the vessel diameter measured at Y3 and Y6, respectively; the arterial pulsatile effect (thin solid lines) was removed by the low-pass filtering (thick solid lines). Minimum–maximum normalization was performed for each low-pass filtered time course, and a time delay (δt) between their peaks was measured [Fig. 2(e)]. The propagation speed of retrograde vasodilation was measured by dividing δy by δt.

**Fig. 3:**
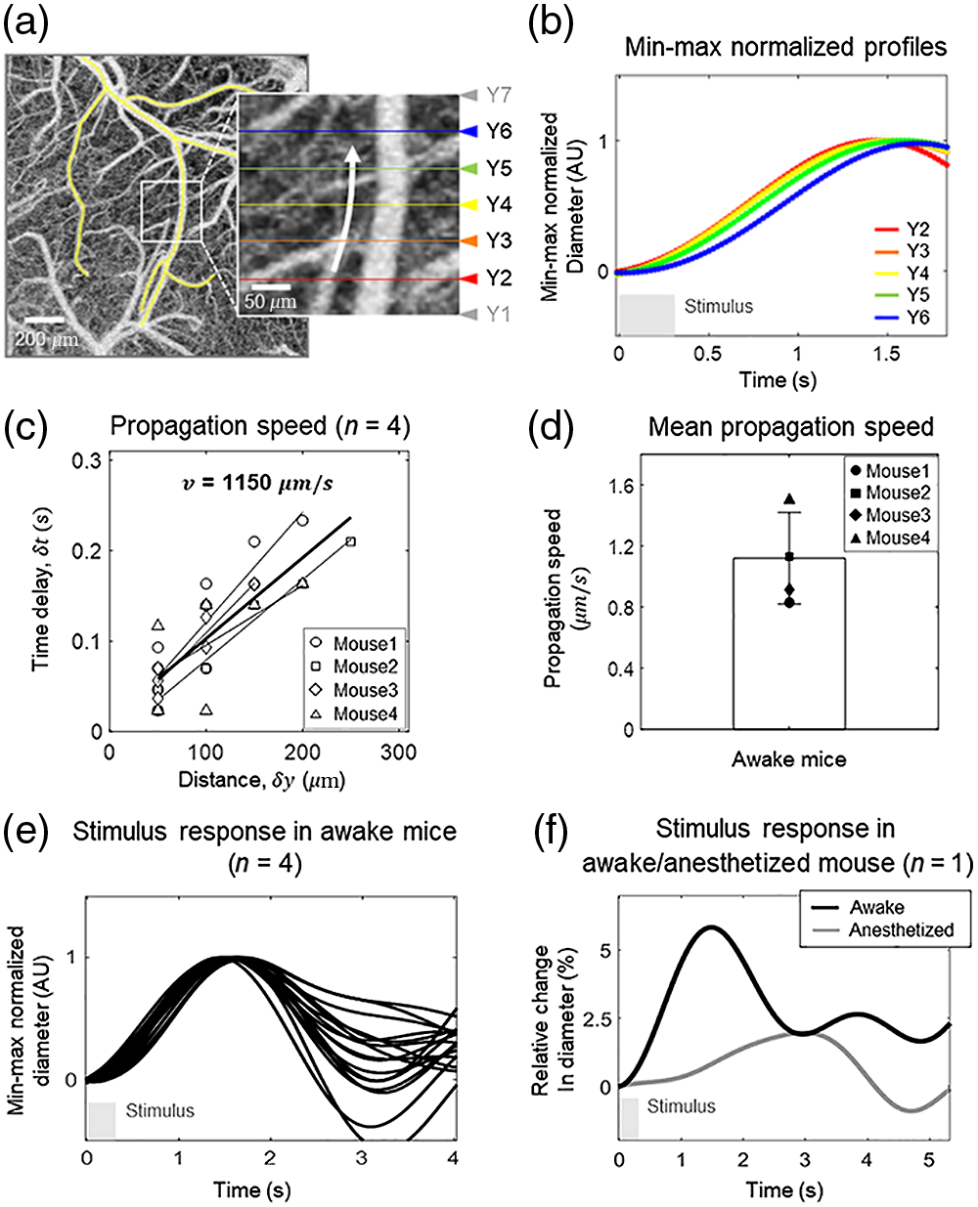
(a) A depth-projected OCT angiogram showing a pial artery and its first-order branches shaded in yellow (0 to 400  μm from the cortical surface, maximum projection). An magnified view of the region where OCTA-2 was performed is indicated by the white square. The horizontal lines indicate the locations where data were acquired. (b) Average time courses of the change in diameter of the pial artery measured at five locations (Y2 to Y6), as indicated in (a). Minimum–maximum normalization was performed for each profile. (c) Scatter plots between δy, which is the distance between two measurement locations, and δt, which is the peak-to-peak delay between the time courses in the four mice. Different animals are indicated with different shaped dots. The regression line for each animal is indicated by a thin solid line. The thick solid line indicates the regression fit of all data. (d) The mean propagation speed of vasodilation for all animals. (e) Average time courses of the diameter of four pial arteries from four mice. For each artery, all time courses of vessel diameter used to obtain the data in (c) are plotted. (f) Hemodynamic responses of the pial artery to the stimulus in anesthetized and awake conditions acquired at the same location in the same animal (Video 3, MP4, 1.7 MB [URL: https://doi.org/10.1117/1.NPh.7.3.030502.3]).

Figure 3(a) shows a depth-projected OCT angiogram of another mouse brain; the white square indicates the region where OCTA-2 was performed. The white arrow in the magnified view of the corresponding region indicates the direction of the propagation of total hemoglobin response, which was identified by OISI (Fig. S3 in the Supplementary Material). Figure 3(b) shows the average time courses of the vessel diameter measured at five different locations (Y2 to Y6), which are indicated by horizontal lines with different colors in Fig. 3(a). As shown in Fig. 3(b), the hemodynamic response was delayed with respect to the response measured at Y2 as the distance δy increases. The propagation speed of vasodilation was measured in four pial arteries from four mice [Fig. 3(c)]. The propagation speed was obtained for each animal using data obtained at different locations (which generate multiple pairs of δy and δt values) where the vessel boundaries were clearly identified. The propagation speed was measured as the slope of the regression line between δy and δt for each animal. The mean propagation speed of vasodilation for all animals was 1.15  mm/s [Fig. 3(d)]. Note that the time difference of 0.01 s between the B-scan acquisitions at Y1 and Y6 due to the sequential imaging of a series of B-scans during the slow axis scan is sufficiently small limiting the error caused by this time difference to be negligible.

Figure 3(e) shows the hemodynamic responses of four pial arteries from four mice. As shown in Fig. 3(e), the hemodynamic responses of the pial arteries were quite similar, especially during the initial dilation. The diameter of the vessels continuously increased and reached the maximum 1.3 to 2 s after the onset of the 0.3-s stimulus. The mean increase in the amplitude of the vasodilation was 7.5% of the baseline diameter. However, the hemodynamic response was altered under anesthesia as shown in Fig. 3(f). The mean change in diameter of the pial artery was smaller and slower in the anesthetic state (indicated by gray solid line) than in the awake state (indicated by black solid line).

## 4 Discussion

In this study, we propose an imaging method for the measurement of the propagation of vasodilation during functional hyperemia in awake mice. Using 3D-OCT angiograms acquired continuously with a temporal resolution of 0.023 s, the propagation speed of vasodilation was measured by investigating the time delay between the maximum dilations at different locations along each pial artery. Our results showed that the mean propagation speed of vasodilation of the pial artery is 1.15  mm/s. To the best of our knowledge, this is the first study to measure the propagation of vasodilation during functional hyperemia in awake animals.

According to our result and the previous reports, the mean propagation speed of total hemoglobin concentration response measured by OISI ranges from 1.6 to 2.4  mm/s.^4^^,^^9^ Although a comparison between the results of OCTA and OISI should be carefully interpreted because of the differences in the measurement principles, OCTA provides more direct assessment of stimulus-induced changes in vessel diameter. It is also noteworthy that the high temporal resolution of the proposed OCTA imaging scheme provided the measurement of the propagation of vasodilation within a pial artery segment without any branch.

OCTA also enables the measurement of temporal profile and amplitude of the vascular response. Although vascular response depends on various factors, such as the location with respect to the activation site, vascular branching structure, and vessel size,^14^^,^^22^ our result showed that pial arteries with different locations and sizes in different animals have a relatively identical stimulus–response profile, especially during the initial dilation process. Furthermore, we showed an altered vascular response in an anesthetized condition.^23^^,^^24^ However, a detailed investigation on alterations in the propagation of vasodilation and other characteristics of hemodynamic responses under various anesthetized conditions is warranted.

In conclusion, our proposed imaging method has a high spatial and temporal resolution and enables label-free measurement of stimulus-induced propagation of vasodilation in awake mice. Our results suggest that the method could be highly useful in the longitudinal monitoring of functional hemodynamic changes with a high spatiotemporal resolution in normal and diseased animal models.

## 5 Appendix

Intensity OCT and OCTA B-scans acquired continuously at the same spatial location appear in Video 2, which shows intensity changes in the OCT B-scans. By computing the cross decorrelation between a pair of neighboring OCT B-scans (variations in a pair of consecutive intensity OCT B-scans), a single cross-sectional OCT angiogram was obtained. As shown in the series of intensity OCT B-scans and OCTA B-scans in Video 3, areas occupied by tissues other than vessels show almost no intensity change during the imaging of a series of B-scans and therefore show almost no OCTA signals. This indicates that the motion artifact occurred during the inter-B-scan time interval was negligibly small. The inter-B-scan time interval used for the study was 1.66 ms.

## Code, Data, and Materials Availability

The data that support the findings of this study are available from the corresponding author, WYO, upon reasonable request.

## References

[1] Girouard H. Iadecola C., “Neurovascular coupling in the normal brain and in hypertension, stroke, and Alzheimer disease,” J. Appl. Physiol. 100(1), 328–335 (2006). 10.1152/japplphysiol.00966.2005 16357086

[r2] Iadecola C., “Regulation of the cerebral microcirculation during neural activity: is nitric-oxide the missing link,” Trends Neurosci. 16(6), 206–214 (1993). TNSCDR 0166-2236 10.1016/0166-2236(93)90156-G 7688160

[3] Iadecola C., “The neurovascular unit coming of age: a journey through neurovascular coupling in health and disease,” Neuron 96(1), 17–42 (2017). NERNET 0896-6273 10.1016/j.neuron.2017.07.030 28957666 PMC5657612

[4] Chen B. R. et al., “A critical role for the vascular endothelium in functional neurovascular coupling in the brain,” J. Am. Heart Assoc. 3(3) (2014). 10.1161/JAHA.114.000787 PMC4309064 24926076

[5] Longden T. A. et al., “Capillary K+-sensing initiates retrograde hyperpolarization to increase local cerebral blood flow,” Nat. Neurosci. 20(5), 717 (2017). NANEFN 1097-6256 10.1038/nn.4533 28319610 PMC5404963

[6] Ogawa S. et al., “Intrinsic signal changes accompanying sensory stimulation—functional brain mapping with magnetic-resonance-imaging,” Proc. Natl. Acad. Sci. U. S. A. 89(13), 5951–5955. 10.1073/pnas.89.13.5951 PMC402116 1631079

[7] Iadecola C. et al., “Local and propagated vascular responses evoked by focal synaptic activity in cerebellar cortex,” J. Neurophysiol. 78(2), 651–659 (1997). JONEA4 0022-3077 10.1152/jn.1997.78.2.651 9307102

[8] Sheth S. A. et al., “Spatiotemporal evolution of functional hemodynamic changes and their relationship to neuronal activity,” J. Cereb. Blood Flow Metab. 25(7), 830–841 (2005). 10.1038/sj.jcbfm.9600091 15744249

[9] Chen B. R. et al., “High-speed vascular dynamics of the hemodynamic response,” Neuroimage 54(2), 1021–1030 (2011). NEIMEF 1053-8119 10.1016/j.neuroimage.2010.09.036 20858545 PMC3018836

[10] Kleinfeld D. et al., “Fluctuations and stimulus-induced changes in blood flow observed in individual capillaries in layers 2 through 4 of rat neocortex,” Proc. Natl. Acad. Sci. U. S. A. 95(26), 15741–15746 (1998). 10.1073/pnas.95.26.15741 9861040 PMC28114

[11] Denk W. Strickler J. H. Webb W. W., “Two-photon laser scanning fluorescence microscopy,” Science 248(4951), 73–76 (1990). SCIEAS 0036-8075 10.1126/science.2321027 2321027

[12] Cai C. et al., “Stimulation-induced increases in cerebral blood flow and local capillary vasoconstriction depend on conducted vascular responses,” Proc. Natl. Acad. Sci. U. S. A. 115(25), E5796–E5804 (2018). 10.1073/pnas.1707702115 29866853 PMC6016812

[r13] Rungta R. L. et al., “Vascular compartmentalization of functional hyperemia from the synapse to the pia,” Neuron 101(4), 762 (2019). NERNET 0896-6273 10.1016/j.neuron.2019.01.060 30790540 PMC6389590

[14] Shih A. Y. et al., “Two-photon microscopy as a tool to study blood flow and neurovascular coupling in the rodent brain (vol 32, pg 1277, 2013),” J. Cereb. Blood Flow Metab. 33(2), 319–319 (2013). 10.1038/jcbfm.2012.177 PMC3390800 22293983

[15] Lee J. et al., “Statistical intensity variation analysis for rapid volumetric imaging of capillary network flux,” Biomed. Opt. Express 5(4), 1160–1172 (2014). BOEICL 2156-7085 10.1364/BOE.5.001160 24761298 PMC3986000

[r16] Radhakrishnan H. Srinivasan V. J., “Compartment-resolved imaging of cortical functional hyperemia with OCT angiography,” Biomed. Opt. Express 4(8), 1255–1268 (2013). BOEICL 2156-7085 10.1364/BOE.4.001255 24009990 PMC3756578

[17] Shin P. et al., “Quantitative hemodynamic analysis of cerebral blood flow and neurovascular coupling using optical coherence tomography angiography,” J. Cereb. Blood Flow Metab. 39(10), 1983–1994 (2019). 10.1177/0271678X18773432 29757059 PMC6775585

[18] Reed M. W. Miller F. N., “Importance of light dose in fluorescent microscopy,” Microvasc. Res. 36(1), 104–107 (1988). MIVRA6 0026-2862 10.1016/0026-2862(88)90042-8 3185298

[19] Saetzler R. K. et al., “Intravital fluorescence microscopy: impact of light-induced phototoxicity on adhesion of fluorescently labeled leukocytes,” J. Histochem. Cytochem. 45(4), 505–513 (1997). JHCYAS 0022-1554 10.1177/002215549704500403 9111229

[20] Goldey G. J. et al., “Removable cranial windows for long-term imaging in awake mice,” Nat. Protoc. 9(11), 2515–2538 (2014).1754-2189 10.1038/nprot.2014.165 25275789 PMC4442707

[21] Moult E. et al., “Ultrahigh-speed swept-source OCT angiography in exudative AMD,” Ophthal. Surg. Lasers Imaging Retina 45(6), 496–505 (2014). RETIDX 0275-004X 10.3928/23258160-20141118-03 PMC4712918 25423628

[22] Devor A. et al., “Stimulus-induced changes in blood flow and 2-deoxyglucose uptake dissociate in ipsilateral somatosensory cortex,” J. Neurosci. 28(53), 14347–14357 (2008). JNRSDS 0270-6474 10.1523/JNEUROSCI.4307-08.2008 19118167 PMC2655308

[23] Franceschini M. A. et al., “The effect of different anesthetics on neurovascular coupling,” Neuroimage 51(4), 1367–1377 (2010). NEIMEF 1053-8119 10.1016/j.neuroimage.2010.03.060 20350606 PMC2879067

[24] Masamoto K. Kanno I., “Anesthesia and the quantitative evaluation of neurovascular coupling,” J. Cereb. Blood Flow Metab. 32(7), 1233–1247 (2012). 10.1038/jcbfm.2012.50 22510601 PMC3390804

