# Supplementary material for: High-speed optical coherence tomography angiography for the measurement of stimulus-induced retrograde vasodilation of cerebral pial arteries in awake mice
Source: Neurophotonics. 2020 Sep 10;7(3):030502. doi: 10.1117/1.NPh.7.3.030502 (PMC7481125; doi:10.1117/1.NPh.7.3.030502)
Supplement: Supplementary file 1 [file NPh_007_030502_SD001.pdf]

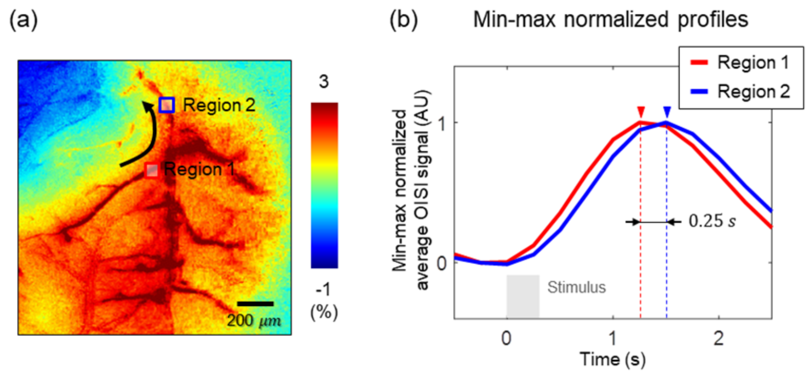

Figure S1. (a) An OISI image acquired 1.25 s after the stimulus onset. The black arrow indicates the direction of the propagation. (b) Changes in the average signal intensity of regions 1 and 2 in (a). Each time course was low-pass filtered with a cutoff frequency of 0.7 Hz and minimum–maximum normalized. The time delay between the peaks of two time courses was 0.25 s. The propagation speed of total hemoglobin concentration response was measured by dividing the distance between two regions (400 μm) by 0.25 s, which is equal to 1.6 mm/s. The measurement was performed in four mice. For each mouse, the distance between two measurement regions was adjusted so that the time delay between the time courses at those regions could be resolved with a temporal resolution of 0.25 s.

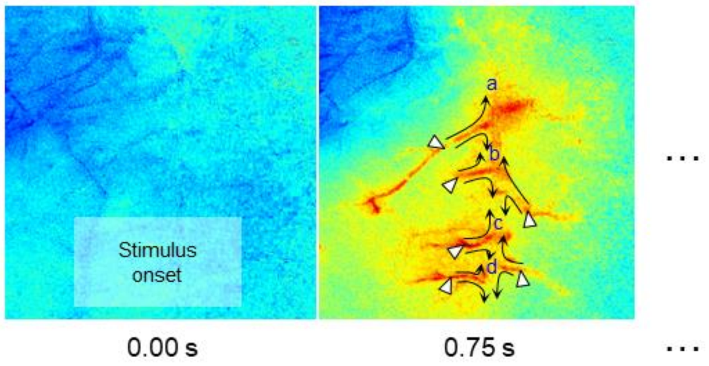

Figure S2. The OISI images from Figure 2(b). Arterioles showing prominent responses in the OISI image are indicated by white arrowheads. Black arrows indicate all possible directions of the propagations from individual arterioles. For each arterial segment b, c, and d, vasodilation could propagate from both sides of the each vessel segment. In the vessel segment a, the direction of signal propagation can be more clearly identified as the signal can propagate in one direction.

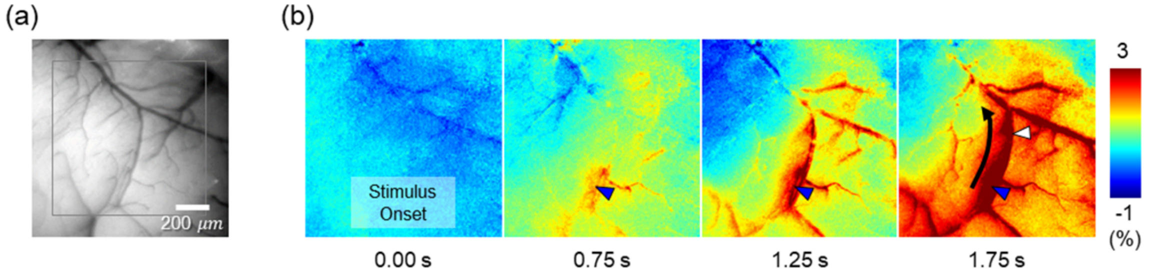

Figure S3. (a) A CCD image of the mouse cranial window. (b) OISI images of the region in (a), showing the propagation of hemodynamic response from the arteriole (blue arrowhead) to the upstream pial artery (white arrowhead). The black arrow indicates the direction of the propagation.
